# Mental Health Help-Seeking Intentions and Preferences of Rural Chinese Adults

**DOI:** 10.1371/journal.pone.0141889

**Published:** 2015-11-06

**Authors:** Yu Yu, Zi-wei Liu, Mi Hu, Hui-ming Liu, Joyce P. Yang, Liang Zhou, Shui-yuan Xiao

**Affiliations:** 1 Department of Social Medicine and Health Management, School of Public Health, Central South University, Changsha City, Hunan Province, China; 2 Department of Psychology, University of Washington, Seattle, Washington, United States of America; University of Stellenbosch, SOUTH AFRICA

## Abstract

**Purpose:**

We aimed to investigate mental health help-seeking intentions and preferences of rural Chinese adults and determine predictors of the intentions.

**Methods:**

A total of 2052 representative rural residents aged 18–60 completed a cross-sectional survey by face-to-face interviews. The survey included seven questions asking about respondents’ help-seeking intentions and preferences, and a series of internationally validated instruments to assess self-perceived health status, depression, anxiety, alcohol abuse, mental health literacy, and attitudes towards mental illness.

**Results:**

Nearly 80% of respondents were willing to seek psychological help if needed, and 72.4% preferred to get help from medical organizations, yet only 12% knew of any hospitals or clinics providing such help. A multivariate analysis of help-seeking intention revealed that being female, having lower education, higher social health, higher mental health knowledge, and physical causal attribution for depression were positive predictors of help-seeking intention.

**Conclusion:**

A huge gap exists between the relatively higher intention for help-seeking and significantly lower knowledge of helpful resources. Predictors of help-seeking intention for mental problems in the current study are consistent with previous studies. Interventions to increase help-seeking for mental problems by Chinese rural adults may be best served by focusing on increasing public awareness of help sources, as well as improving residents’ mental health literacy and social health, with special focus on males and those more educated.

## Introduction

Untreated mental illness is a significant global health issue. Up to 30% of the world’s population is suffering from mental illness, yet over two-thirds of them receive no treatment [1–3]. In the United States, mental illness affects 31% of the population every year, of whom 61% are untreated [3,4]. The untreated mental illness rate is 60% in Australia [5] and 74% in Europe [6]. In China, over 90% of the 170 million adults with mental disorders are not treated [7]. Untreated mental illness leads to worse health outcomes, which incur huge economic and social losses [8–10]. It is thus important to understand barriers impeding people from seeking help for mental health problems in order to implement targeted interventions to improve the well-being of those affected.

In addition to structural barriers such as shortage of infrastructure, lack of qualified doctors, financial hardship, and limited access to mental healthcare providers that prevent people from seeking mental health services [7,11], a series of individual-level factors also influence people’s help-seeking intentions and behaviors. A literature review of these individual-level factors has produced three major categories: socio-demographics, health status, and mental health literacy, as described below. We have included representative variables from each of these three categories in our data analysis.

### Socio-demographics and help-seeking

Socio-demographic differences in help-seeking have been extensively established in the literature, with gender being the most consistently reported significant factor predicting people’s help-seeking. A growing body of research has congruously indicated that females of all age groups have more favorable intentions than males for seeking mental health services and are more likely to engage in professional help-seeking behaviors when confronted with psychological distress [10,12–18]. Education is also a well-documented predictive factor for people’s help-seeking, with accumulating evidence showing that higher education is associated with higher intentions and behaviors for mental health problems regardless of ethnicity and country of research [17,19,20]. Evidence for the association between age and help-seeking, however, is conflicting, with most studies showing positive associations between increasing age and help-seeking intentions and behaviors [10,13,17,18,21], although a few reported negative [12,22,23] or no associations [24]. Furthermore, other socio-demographic variables including ethnic background, income, and marital status have also been reported to influence people’s help-seeking for mental problems in various studies with inconsistent findings [12,13,16–18,22,23,25].

### Health status and help-seeking

Health status, especially mental health status, has been extensively reported to be associated with help-seeking intentions and behaviors, although also with inconsistent results. The majority of literature suggests that poorer mental health status is associated with increased mental health service use, evidenced by a large body of studies showing that those having functional impairment [16], meeting criteria for one or two mental disorders such as depression, anxiety [10,12,16], and substance abuse [21], and/or with higher self-perceived mental problems or psychological distress [15,26] are more likely to seek help from both formal and informal sources. However, one study showed that a reluctance to seek help was characterized by high mental symptoms and low satisfaction with one’s own mental health [17], while another study reported no significant association between mental health and help-seeking intention [27]. One cohort study found that the association between having a mental disorder and help-seeking varies according to the help source, with a positive association between depression and seeking help from family and friends, but no association between depression and seeking help from a healthcare professional [28].

### Mental health literacy and help-seeking

Mental health literacy (MHL) refers to knowledge and beliefs about mental health disorders that aid in recognition, management, or prevention [29]. It mainly includes three aspects including recognition of mental disorders, knowledge of factors relating to mental health, and attitudes and beliefs about mental disorders [30]. MHL has been one of the most studied areas related to mental health help-seeking, with accumulating evidence showing that people with lower MHL are less likely to seek help for mental problems. Of the various aspects of mental health literacy, recognition of mental disorders emerges as an initial and important factor, as it is the first step to seeking help from professional sources [31,32]. Numerous studies have documented that inability to recognize mental disorders is associated with delay in and reduced likelihood of help-seeking [31–33]. Knowledge about mental health is another factor that has been frequently reported to be a predictive factor for help seeking for mental illness, with lack of mental health knowledge identified in the literature as an important barrier impeding people from seeking treatment [17,34]. Among the various aspects of attitudes towards mental disorders, social stigma is the most researched area, with stigma and shame being reported as major challenges people face when seeking help for mental problems [17,22,32,34]. Indeed, a recent meta-analysis of quantitative and qualitative studies reported a small- to moderate-sized negative effect of stigma on help-seeking [35].

A review of existing literature on help-seeking for mental problems reveals two major limitations. First, most of the studies were conducted in developed countries such as the US, Australia, and Europe; knowledge about help-seeking in developing countries such as China is scarce, even less is known about the rural areas in China. Second, most of the literature focused on one or two factors of the above-mentioned aspects, an exhaustive study exploring the influence of all the above-mentioned factors on help-seeking is lacking.

The main objective of the present study is to conduct a cross-sectional study with a representative Chinese rural sample, investigating the intentions and preferences of rural Chinese adults towards seeking help for mental health problems. In addition to the help-seeking information, we aimed to conduct exploratory, multivariate analyses of the data in seeking to predict help-seeking intention, based on the theoretical framework of the above-mentioned three major types of influencing factors.

## Materials and Methods

### Participants

This investigation is based on the 2010 National Science and Technology Support Program, “The Assessment, Warning, and Intervention Study on the Emotional Problems of the Chinese Population”, a large psychosocial epidemiological study on emotional distress of rural residents. The survey was conducted from November 2010 to August 2011 with 2052 permanent residents aged 18–60 living in the rural areas of Liuyang county, Hunan province. In order to get a sample that is as representative of all rural residents of Liuyang city as possible, a multistage cluster-sampling method was adopted to identify subjects. Two towns (Gaoping and Yong’an) were randomly selected from 33 towns of Liuyang county, and then two villages (Shiwan and Ma’an from Gaoping; Lutang and Shuishan from Yong’an) were randomly selected from each town, leading to a total sampling frame of 4 villages that were representative of rural populations in Liuyang, in terms of geography, socio-demographics, mental health care access and outreach activities. 2158 residents were selected as the final sample. Eligible participants were required to be residents living in the rural areas for more than half a year, who were aged 18 to 60. Those who were not living in the areas during the research period, had difficulty in communication due to serious physical or mental illness, or were cognitively impaired or actively psychotic were excluded. The response rate was 95%, resulting in a sample of 2052 individuals.

The majority of the participants were married (91%), non-religious (90%), and with an educational level of middle school or below (85%). 61% of the sample was employed, and over two-thirds had a monthly income of lower than 300 RMB. Gender was relatively evenly split in the sample with 56% female and 44% male. Age of the participants ranged from 18 to 59, with a median of 42.

### Procedures

Ethics approval was granted by the Ethics Review Committee of the School of Public Health of Central South University. Interviewers visited each household and explained the purpose and process of the study to the participants. After providing written informed consent, each eligible respondent was invited to complete a series of questionnaires (see measures below) by face-to-face interviews. The answers were checked by a quality control person to ensure that there were no inconsistencies or missing items. All participants were reimbursed with some small gifts such as kitchen utensils (RMB ¥ 12) in return for their participation.

### Constructs and Measures

#### Help Seeking

Help seeking intention was assessed by the question, “If you had severe psychological or mental health problems, would you seek help from professional sources (e.g., counselor, psychologist) for them?” Help seeking preference was assessed by the question, “When you have mental health problems, what mental healthcare organization would you most likely seek help from?” Based on previous studies in the field [10,18,36,37], we also incorporated more information related to help seeking intention and preference, including reasons for *not* seeking help, self-shaming attitudes towards seeking help, favored approaches to mental health education and service, and awareness of potential help resources. Detailed information about the questions and optional answers can be seen in the S1 Appendix.

#### Health Status

*SRHMS*. The Self-Rated Health Measurement Scale (SRHMS) is a 48-item scale developed and revised by Xu et al. [38,39] to assess self-rated health in both hospitalized and general populations. It includes three main subscales measuring physical health, mental health, and social health. Each item is rated on a 10-point scale from 1 = "extremely poor health" to 10 = "extremely good health". The SRHMS has widespread use in China [38,40,41] and demonstrated good internal consistency in the current study, with a Cronbach’s α coefficient of 0.91.

*PHQ-9*. Symptoms of depression were measured using the Patient Health Questionnaire (PHQ-9) [42], a nine-item screening tool based on criteria for depressive disorders in the Diagnostic and Statistical Manual of Mental Disorders (DSM-IV) [43]. Respondents are asked whether they have experienced 9 symptoms in the past two weeks on a 4-point Likert scale from 0 = "not at all", to 3 = "nearly every day". The total score ranges from 0 to 27, with scores of 5, 10, 15, and 20 representing cut-offs for mild, moderate, moderately severe, and severe depression, respectively. A meta-analysis of the scoring method of the PHQ-9 showed that a cut-off point of ≥10 has the best diagnostic performance and is thus used in the current study to differentiate people with and without depression [44]. The Chinese version of the PHQ-9 has been well validated in multiple studies [45–47] and demonstrated good internal consistency in the current study, with a Cronbach’s α coefficient of 0.81.

*GAD-7*. Symptoms of anxiety were measured using the Generalized Anxiety Disorder Scale (GAD-7), a 7-item self-report scale developed by Spitzer et al. [48] to assess symptoms of and screen for generalized anxiety. Respondents are asked to choose how often they have been bothered by anxiety symptoms on a 4-point Likert scale from 0 = "not at all" to 3 = "nearly every day". The total score ranges from 0 to 21, with a score of ≥10 representing the optimum cut-off for screening for anxiety disorders [49]. The Chinese version of the GAD-7 has been widely used and well validated in multiple studies [50,51], and has demonstrated good internal consistency in the current study, with a Cronbach’s α coefficient of 0.88.

*AUDIT*. Alcohol use disorders were measured by the alcohol use disorders identification test (AUDIT), a 10-item scale developed by the World Health Organization (WHO) [52] to identify hazardous and harmful drinking in diverse settings and multicultural populations [53]. All item scores range from 0 to 4 and the total score ranges from 0 to 40, with a score of ≥8 representing the optimal cut-off for hazardous drinking. The Chinese version of the AUDIT has been widely used and well validated in multiple studies [54] and demonstrated good internal consistency in the current study, with a Cronbach’s α of 0.67.

#### Mental Health Literacy (MHL)

*Recognition of Depression*. Recognition of depression was assessed using a vignette (S2 Appendix) drawn from Jorm et al. [29] depicting a person with classical depressive symptoms meeting DSM-IV diagnostic criteria. Participants were asked a multiple choice question to describe what was going on in the vignette, with several mental health disorders as answer choices. A second multiple choice question assessed for attribution of the symptoms described in the vignette.

*MHKQ*. Mental health knowledge was assessed using the Mental Health Knowledge Questionnaire (MHKQ), a 20-item scale developed by the Chinese Ministry of Health (MOH) in 2009 to assess public knowledge and awareness of mental health. The 20 items are shown in S3 Appendix, with items 1–16 representing statements about mental health and items 17–20 asking about the four international mental health promotion days such as International Mental Health Day and World Sleep Day. Correct answers are given one point resulting in a total score ranging from 0 to 20 with a higher score representing higher levels of mental health knowledge. Past psychometric testing of the scale has reported internal consistency (Cronbach’s α coefficients) ranging from 0.57 to 0.73 [55–57] and a 2-week test–retest reliability as measured by intra-class correlation coefficients of 0.68 [57]. In the present study, Cronbach’s α = 0.61.

*PDD*. Attitudes towards mental illness were investigated by using the Perceived Devaluation–Discrimination Questionnaire (PDD), a 12-item scale developed by Link et al. [58,59] to assess negative beliefs about patients with mental illness. Respondents are asked to choose how much they agree with each statement on a 4-point Likert scale from 0 = "strongly disagree" to 4 = "strongly agree" [59,60]. The total score ranges from 12 to 48, with a higher score indicating more negative attitudes towards mental illness. The PDD has been widely used and well validated in multiple studies [59,61] and demonstrated good internal consistency in the current study, with a Cronbach’s α of 0.67.

### Statistical analysis

Data were analyzed using STATA software version 12.0. Scales and indices were tested for reliability. Exploratory and summary statistics were obtained for all variables within the dataset. Data were examined for the presence of missing values, influential values and outliers, skew, and kurtosis. For the question of help-seeking intention, those answering “definitely yes” and “probably yes” were merged into a “yes” group, while those answering “definitely no” and “probably no” were merged into a “no” group. For the question of shame regarding help-seeking behaviors, those answering “very embarrassed” and “a little embarrassed” were merged into an “embarrassed” group, while those answering “not very embarrassed” and “definitely not embarrassed” were merged into a “not embarrassed” group. Both univariate and multivariate logistic regression analyses were conducted to examine predictors of help-seeking intention.

## Results

### Help-seeking intentions and preferences

Table 1 shows the results of the respondents’ help-seeking intentions and preferences for mental problems. Nearly 80% of respondents indicated that they would seek help from a professional if they experienced a severe psychological problem. Of this 80%, nearly 60% reported that they would not feel ashamed if their help-seeking behaviors were disclosed to their friends and relatives. 72.4% respondents chose medical institutions as their first-choice mental healthcare organization, yet only 12.2% knew of any hospitals or clinics in their neighborhood providing psychological help. The first choice approaches for mental health services were almost equally distributed among the 12 option responses, with lectures, brochures, and hotlines chosen slightly more often than the other options. Although 12% respondents preferred to receive psychological help through hotlines, only 2.1% knew of any psychological hotlines. Regarding reasons for not seeking help, among the 318 respondents who expressed reluctance to seek help for mental health problems, the most commonly endorsed reason was “I want to solve it myself”, followed by “concern about the cost”, and “don’t know where to get help”.

**Table 1:** Help-seeking intentions and preferences of rural Chinese adults (*N* = 2052).

Variable	*n*	%
Professional help-seeking behavior (*N* = 2052)		
Yes	1638	79.8
No	318	15.5
Unknown	96	4.7
Will you feel ashamed if your help-seeking behaviors were disclosed to your relatives and friends? (*n* = 1638)		
Yes	625	38.2
No	976	59.6
Unknown	37	2.2
Top five reasons for not seeking help (*n* = 318)		
Want to solve it on one’s own	271	85.2
Concern about the cost	139	43.7
Don’t know where to get help	113	35.5
Take too much time or inconvenient	112	35.2
Think treatment is ineffective	103	32.4
First-choice organization for mental health service (*N* = 2052)		
Medical institution	1485	72.4
Governmental organization	253	12.3
Private institution	111	5.4
Educational department	93	4.5
Non-governmental organizations(NGO)	17	0.8
Other	93	4.5
Top five preferred approaches for mental health knowledge and service access (*N* = 2052)		
Lectures	394	19.2
Brochures	248	12.1
Hotline	246	12.0
Bulletin Boards	163	7.9
Individual counseling	143	7.0
Do you know of any hospital or clinic in your neighbor that can solve psychological problems? (*N* = 2052)		
No	1802	87.8
Yes	250	12.2
Do you know of any psychological hotlines? (*N* = 2052)		
No	2010	97.9
Yes	42	2.1

### Predictors of help-seeking intentions

The results of a univariate logistic regression of the effect of socio-demographics, health status, and MHL on the respondents’ willingness to seek psychological help for mental problems is presented in Table 2. Factors that were significantly associated with respondents’ help-seeking intention in bivariate analyses were: gender, education, self-perceived health, depression, anxiety, recognition of depression, and mental health knowledge. Being female, having better self-perceived health including physical, mental, and social health, higher mental health knowledge, and attribution of the depression vignette to a physical problem or over-fatigue positively influenced help-seeking intention, while higher education, having anxiety and depression, and correct recognition of depression negatively influenced help-seeking.

**Table 2:** Univariate analyses by using univariate logistic regression of socio demographics, MHL, health status, and professional help-seeking behaviors (*n* = 1956).

	Professional help-seeking intention (*n* = 1956)			
Variable	No (*n* = 318)*	Yes (*n* = 1638) *	*OR* (95% CI)	*p*-value
**Socio-demographics**				
Location				
Lutang	106 (33.33)	501 (30.59)		
Shuishan	53 (16.67)	344 (21.00)	1.37 (0.96, 1.96)	
Ma’an	87 (27.36)	418 (25.52)	1.02 (0.74, 1.39)	
Shiwan	72 (22.64)	375 (22.89)	1.10 (0.79, 1.53)	0.325
Age (y, mean±SD)	41.09±0.60	41.75±0.26	1.01 (0.99, 1.02)	0.317
Gender				
Male	164 (51.57)	699 (42.67)		
Female	154 (48.43)	939 (57.33)	**1.43 (1.12, 1.82)**	0.004
Education				
Primary school or less	106 (33.33)	672 (41.03)	ref	
Middle school	158 (49.69)	722 (44.08)	**0.72 (0.55, 0.94)**	0.016
High school and above	54 (16.98)	244 (14.90)	0.71 (0.50, 1.02)	0.064
Occupation				
Unemployed	128 (40.25)	627 (38.30)	ref	
Employed	190 (59.75)	1010 (61.70)	0.92 (0.72, 1.18)	0.514
Income (RMB/month)				
150 or less	136 (44.59)	748 (47.07)	ref	
151–300	74 (24.26)	379 (23.85)	0.93 (0.68, 1.27)	0.651
300 or greater	95 (31.15)	462 (29.07)	0.88 (0.66, 1.18)	0.400
Marriage				
Married/cohabited	286 (89.94)	1494 (91.21)	ref	
Never married	25 (7.86)	111 (6.78)	0.85 (0.54, 1.34)	0.481
Divorced/separated	7 (2.20)	33 (2.01)	0.90 (0.40, 2.06)	0.807
Religion				
No	290 (91.19)	1471 (89.80)	ref	
Yes	28 (8.81)	167 (10.20)	0.85 (0.56, 1.29)	0.449
**Health status**				
HRHMS(mean±SD)				
Physical health	8.16 ± 0.07	8.43 ± 0.03	**1.23 (1.11, 1.36)**	**<0.01**
Mental health	7.50 ± 0.09	7.87 ± 0.03	**1.19 (1.10, 1.29)**	**<0.01**
Social health	6.74 ± 0.09	7.18 ± 0.04	**1.19 (1.11, 1.28)**	**<0.01**
Total health	7.49 ± 0.07	7.85 ± 0.03	**1.33 (1.20, 1.48)**	**<0.01**
Depression				
No	273 (85.85)	1510 (92.19)	ref	
Yes	45 (14.15)	128 (7.81)	**0.51 (0.36, 0.74)**	**<0.01**
Anxiety				
No	288 (90.57)	1546 (94.38)	ref	
Yes	30 (9.43)	92 (5.62)	**0.57 (0.37, 0.88)**	**0.011**
Alcohol abuse				
No	280 (88.05)	1477 (90.17)	ref	
Yes	38 (11.95)	161 (9.83)	0.80 (0.55, 1.17)	0.253
**MHL**				
Knowledge (mean±SD)	11.37 ± 0.18	11.74 ± 0.07	**1.04 (1.00, 1.09)**	0.037
Recognition of depression				
Wrong	255 (80.19)	1404 (85.71)	ref	
Right	63 (19.81)	234 (14.29)	**0.67 (0.50, 0.92)**	0.012
Attribution of depression				
Psychological problem	204 (64.15)	924 (56.41)	ref	
Physical problem	46 (14.47)	348 (21.25)	**1.67 (1.19, 2.35)**	0.003
May be bewitched	5 (1.57)	14 (0.85)	0.62 (0.22, 1.74)	0.361
Over fatigue	49 (15.41)	311 (18.99)	**1.40 (1.00, 1.96)**	0.050
Other	14 (4.40)	41 (2.50)	0.65 (0.35, 1.21)	0.172
Attitude toward mental illness (mean±SD)	29.85 ± 0.27	30.27 ± 0.14	1.01 (0.99, 1.04)	0.207

* means n (%) if not otherwise indicated.

A further multivariate logistic regression was conducted to determine the predictors of help-seeking intention. Among the 16 factors that were included in the model, five factors remained significant after controlling for all the other factors, including gender, education, perceived social health, mental health knowledge, and physical attribution for the depression vignette (Table 3). Females were more likely to seek psychological help for mental problems than males with an odds ratio of 1.5. Better perceived social health and higher mental health knowledge were associated with increased likelihood of help-seeking, while higher education was associated with reluctance to seeking help. Interestingly, physical attribution for the depression vignette was the strongest predictor of help-seeking intention with an *OR* of 1.59 (95%CI = 1.10–2.31). Compared to those who correctly recognized the symptoms in the vignette as originating from a psychological problem, those misattributing the vignette as a physical problem had a 58% increased likelihood of help-seeking.

**Table 3:** Multivariate logistic regression of socio-demographics, MHL, health status and professional help-seeking behaviors (n = 1956)*.

Variable	Professional help-seeking behavior (n = 1956)	
	adjusted OR(95% CI)	P value
Gender		
Male	ref	
Female	**1.49 (1.11, 2.00)**	**0.005**
Education		
Primary school or less	ref	
Middle school	**0.59 (0.43, 0.82)**	**0.002**
High school and above	**0.52 (0.33, 0.83)**	**0.008**
Social health	**1.16 (1.06, 1.28)**	**0.001**
Mental health knowledge	**1.06 (1.01–1.11)**	**0.034**
Attribution of depression		
Psychological problem	ref	
Physical problem	**1.59 (1.10, 2.31)**	**0.015**
May be bewitched	0.57 (0.20, 1.67)	0.296
Over fatigue	1.30 (0.90, 1.88)	0.174
Other	0.70 (0.35, 1.40)	0.286

*Location, age, occupation, income, marital status, religion, depression, anxiety, alcohol abuse, recognition of the depression vignette and attitudes towards mental illness were not significantly associated with help-seeking intention and thus are not listed in this table

## Discussion

### Summary of the findings

The main findings of the study are that 80% of respondents indicated that they would seek help from a professional for psychological problems, and 72% respondents preferred to receive help from medical institutions, yet only 12.2% knew of any hospital or clinic in their neighborhood that provided psychological help. The wide gap between high help-seeking intention and low knowledge of help sources confirms the importance and need to increase awareness and knowledge of resources for mental health. Being female, having better perceived social health, higher mental health knowledge, and physical attribution for the depression vignette were positive predictors for help-seeking intention, while higher education was the only negative predictor for help seeking intention.

### Mental health help-seeking intentions and preferences

Nearly 80% of rural adults in this study indicated that they would seek help for mental health problems, a percentage similar to the 75% reported in a representative Swiss adult sample [17] and 78% in a random national sample in the UK [18], and much higher than the 55.7% reported in a Australian rural adolescent sample [10], 42% in military personnel in the UK [16], and 35.6% in a representative Taiwanese adult sample [62]. Although the high help-seeking intention for mental health problems in this sample is favorable, the low knowledge of potential help sources is discouraging. Despite the availability of over 7 clinics and hospitals in the vicinity and hundreds of national hotlines, only 12.2% knew of any hospital or clinic in their neighborhood providing psychological help, and only 2.1% knew of any psychological hotlines. The huge gap between high help-seeking intention and low knowledge of help sources confirms the importance and need to increase rural residents’ awareness of potential help sources through more public campaign and educational activities.

For those who were not willing to seek help for a mental health problem in this study, wanting to solve it on one’s own was the most frequently raised reason, a rationale likely explainable by cultural values. In Chinese culture, psychological distress is often thought of as a result of personal weakness, lack of willpower, and malingering bad thoughts [63], which would incur shame and stigma if exposed in order to seek help. Therefore, Chinese participants may prefer to solve problems on their own by suppressing their emotional distress instead of seeking external help [37,64,65].

In terms of help-seeking preferences, the first choice of mental health service organization for the majority of participants was a medical institution, and the top three preferred approaches for mental health knowledge and service were through lectures, brochures, and hotlines. One of the implications of this finding is the potential for involving primary care providers and physicians from clinics and hospitals in mental health promotional activities in the rural areas. With adequate training, support, and supervision for health workers, promotion of the public’s mental health literacy and wellbeing though giving lectures, distributing brochures, and setting up hotlines in rural areas may be highly beneficial. Furthermore, alternative help resources such as government organization and private institutions may also be developed and made available to the rural residents to reduce the burden for health providers.

### Predictors for help-seeking intentions

#### Socio-demographics

The finding that females were more likely to seek help for mental health problems than males is consistent with existing literature [10,12–18]. There are several possible reasons including higher rates of mental disorders [66,67], lower levels of stoicism and personal stigma related to mental health problems [14], and more positive attitudes concerning psychological openness [13] in women compared to men, as well as gender-role differences where men’s traditionally advantaged social status and greater power may make it more difficult to seek help for mental health issues [14]. Culture may also be an additional explanation, especially in an Asian culture where men’s masculinity is demonstrated through power, dominance, self-control and self-reliance, expression of psychological distress, and related help-seeking behaviors are regarded as a lack of masculinity and therefore often avoided [64]. One implication of the finding is that males may be targeted for interventions aimed at improving help-seeking intention.

The finding that higher education was negatively associated with intention to seek help is contrary to the majority of studies in the literature showing positive association between higher education and help-seeking [17,19,20]. Importantly, higher education does not necessarily mean higher mental health knowledge, as shown in our results, higher education was associated with decreased likelihood of help-seeking intention, while higher mental health knowledge was associated with increased likelihood of help-seeking intention. Therefore, the unexpected negative association between education and help-seeking intention cannot be explained by MHL, but may be due to the higher level of perceived stigma among those more educated, which may impede them from seeking help, as evidenced by a qualitative study on resistance to help-seeking among medical students. In this group of highly educated college students, viewing mental problems as personal weakness and concern about future career progression were linked to avoidance of appropriate help-seeking [68]. This suggests that more anti-stigma campaigns and activities towards mental illness are needed as well among those with higher education.

#### Health status

Better perceived social health was a positive predictor for help-seeking intention, which may be due to the high perceived social support from their social networks. Social networks act as an important source of advice, information, and support for health issues, in which positive values and beliefs related to help seeking are often transmitted and thus promote help-seeking [23,69,70]. As a result, those with higher perceived social support always exhibited more positive attitudes towards mental health help-seeking and were more likely to seek help and get help for mental health problems [23,32]. This has implications for improving rural social networks in order to increase people’s mental health help-seeking intention.

#### Mental Health Literacy (MHL)

Regarding mental health literacy, higher mental health knowledge was associated with increased likelihood to seek help, which is in accordance with the literature [17,34]. However, no associations were found between attitudes towards mental illness and help-seeking intention in both univariate and multivariate logistic regression in the current study. This absence is contrary to the commonly reported finding that social stigma towards mental illness is a major deterrent to help seeking [17,22,32,34]. However, one study of a nationally representative Taiwanese sample reported a similar lack of association between attitudes toward mental illness and help-seeking preference [62]. Furthermore, attribution of the depression vignette as a physical instead of psychological problem was positively associated with help-seeking intention, which is also supported by previous studies [36,37,62]. This may be explained by the somatization of mental disorders in Chinese culture [37], where psychological distress is often regarded as personal weakness and lack of willpower which requires little medical attention [63], while somatic complaints are often perceived as more important due to the emphasis and amplification of physiological changes and their central role in daily lives [71]. As a result, somatic complaints are more effective and perceived as more legitimate for bringing Chinese people to seek help compared to psychological complaints [37]. In addition, the label of somatic complaints is more often free of the stigma, fear, guilt, or ambivalence that is attached to psychological complaints [72] and thus are more likely to be used to solicit help. An implication of these findings is that mental health literacy may be increased to improve rural residents’ help-seeking intention, with more focus on knowledge than attitude.

### Limitations

One major concern with this study is that the finding of high intention of and low perceived shame for help seeking for mental problems may due to information bias caused by social desirability. Mental illness is highly stigmatized and rarely discussed openly in a Chinese culture [73,74]; it is likely that the rural respondents in the current study may have overstated their openness towards help-seeking for mental problems during the face-to-face interview. Future study may benefit from using a more private interview method such as audio computer-assisted self-interviewing to get a more reliable answer. Another limitation is the cross-sectional design of the study making it impossible to infer causality between the influencing factors and help-seeking intention. In the future, it may be worthwhile to conduct a cohort study to investigate the effect of mental symptomatology and mental health literacy on people’s help-seeking intention and subsequent behaviors. A third limitation is the absence of qualitative exploration of people’s help-seeking preferences for mental health problems, such as whether their preferred types of health providers are general medical doctors or psychologists. Future research may add more detailed questions to get a more comprehensive understanding of people’s help-seeking preferences.

## Conclusions

In conclusion, this study represents an attempt to better understand the help-seeking intentions and preferences of rural Chinese adults. A huge gap exists between high help-seeking intention and low knowledge of help sources, confirming the importance and need to increase rural residents’ awareness and knowledge of possible help sources for mental health through various approaches such as lectures, brochures, and hotlines provided by medical institutions. Furthermore, the positive influence of female gender, higher social health, higher mental health knowledge, and physical causal attribution for depression, as well as the negative influence of higher education on help-seeking intention is informative. Future intervention toward promoting help-seeking may focus on improving residents’ mental health literacy and social health, with special attention to males and the more educated.

## Supporting Information

S1 Appendix Survey on help seeking. (DOCX)

S2 Appendix Vignettes with sample questions and potential response options. (DOCX)

S3 Appendix Mental health knowledge questionnaire. (DOCX)

S1 Data Original dataset. (DTA)

S1 Command Stata command for data analysis. (DO)
