# Supplementary material for: Mental Health Help-Seeking Intentions and Preferences of Rural Chinese Adults
Source: PLoS One. 2015 Nov 6;10(11):e0141889. doi: 10.1371/journal.pone.0141889 (PMC4636424; doi:10.1371/journal.pone.0141889)
Supplement: S1 Appendix — (DOCX) [file pone.0141889.s001.docx]

**S1 APPENDIX: SURVEY ON HELP SEEKING**

1. If you had severe psychological or mental health problems, would you seek help from professional sources (e.g., counselor, psychologist) for them? (Please choose one answer.)

① Definitely yes

② Probably yes

③ Probably no

④ Definitely no

⑤ Not sure/refuse to answer

2. We want to know about why you wouldn’t seek help for mental health problems. Some possible reasons are listed below, please choose whether each reason fits your situation.

| **Reasons** | **Yes** | **No** | **Unsure** |
| --- | --- | --- | --- |
| I want to solve it by myself |  |  |  |
| I don’t know where or who should I seek help from |  |  |  |
| I have other concerns such as transportation difficulty and scheduling conflicts that make me unable to seek help |  |  |  |
| I think it’s too inconvenient and costs too much time |  |  |  |
| I am concerned about the financial cost |  |  |  |
| I don’t have health insurance |  |  |  |
| I don’t think treatment is effective |  |  |  |
| I had received treatment before, but it was ineffective |  |  |  |
| I am concerned about what others will think of me if they know I am seeking treatment |  |  |  |
| I am afraid of being hospitalized involuntarily |  |  |  |
| I am dissatisfied with the current medical services |  |  |  |
| Others |  |  |  |

3. Will you feel embarrassed if other people know that you are seeking help from professional sources for your mental health problems?

① Very embarrassed

② A little embarrassed

③ Not very embarrassed

④ Definitely not embarrassed

⑤ Not sure/refuse to answer

4. Usually when you have mental health problems, what mental healthcare organization would you most likely seek help from?

① Government organizations such as mental health service institutions set up by the women’s association or disability association

② Medical institutions

③ Psychological aids provided by non-government organization

④ Mental health service institutions set up by educational institutions

1. Private mental health service facilities
2. Other

5. If you have emotional distress, psychological problems, or substance use disorders, what’s your most favorable approach to access mental health education and services?

① Psychological bulletin board

② Psychological hotline

③ Internet psychological service

④ Lecture on psychological topic

⑤ Brochures or pamphlet on psychological health

⑥ Individual psychological counselling

1. Group psychological counselling
2. Community mental health education
3. Group discussion and panel discussion
4. Activities organized by self-helpers
5. Activities organized by volunteers
6. Psychological test and screening
7. Psychological records keeping
8. Knowledge test on mental health
9. Psychological crisis intervention
10. Other

6. Do you know of any hospital or clinic in your neighborhood that provides mental health resources?

① Yes

② No

7. Do you know of any psychological hotlines?

① Yes

② No
