# Supplementary material for: Mental Health Help-Seeking Intentions and Preferences of Rural Chinese Adults
Source: PLoS One. 2015 Nov 6;10(11):e0141889. doi: 10.1371/journal.pone.0141889 (PMC4636424; doi:10.1371/journal.pone.0141889)
Supplement: S2 Appendix — (DOCX) [file pone.0141889.s002.docx]

**S2 APPENDIX: VIGNETTES WITH SAMPLE QUESTIONS AND POTENTIAL RESPONSE OPTIONS**

DEPRESSION VIGNETTE

Mr. Wang is 30 years old. He has been feeling unusually sad and miserable for the last few weeks. Even though he is tired all the time, he has trouble sleeping nearly every night. Mr. Wang does not feel like eating and has lost weight. He cannot keep his mind on his work and puts off making decisions. Even day-to-day tasks seem too much for him. This has come to the attention of his boss, who is concerned about Mr. Wang’s lowered productivity.

1. What do you think is wrong with Mr. Wang? Please fill in the item (choose only one) that you think best describes his problem.
2. Physical weakness
3. Neurasthenia
4. Depression
5. Mania
6. Obsessive-compulsive disorder
7. Schizophrenia
8. Others
9. Unknown
10. What do you think is the primary cause of this problem? Please fill in the item (choose only one) that you think best explains his problem.
11. Physical problem
12. Mental problem
13. Possession by evil spirits
14. Over fatigue
15. Others
