# Supplementary material for: Mental Health Help-Seeking Intentions and Preferences of Rural Chinese Adults
Source: PLoS One. 2015 Nov 6;10(11):e0141889. doi: 10.1371/journal.pone.0141889 (PMC4636424; doi:10.1371/journal.pone.0141889)
Supplement: S3 Appendix — (DOCX) [file pone.0141889.s003.docx]

S3 APPENDIX MENTAL HEALTH KNOWLEDGE QUESTIONNAIRE (MHKQ)

| Item | Question | Answer |
| --- | --- | --- |
| 1 | Mental health is a component of health. | Yes |
| 2 | Mental disorders are caused by incorrect thinking | No |
| 3 | Many people have mental problems but do not realize it | Yes |
| 4 | All mental disorders are caused by external stressors | No |
| 5 | Components of mental health include normal intelligence, stable mood, a positive attitude, quality interpersonal relationships, and adaptability | Yes |
| 6 | Most mental disorders cannot be cured | No |
| 7 | Psychological or psychiatric services should be sought if one suspects the presence of psychological problems or a mental disorder. | Yes |
| 8 | Psychological problems can occur at almost any age | Yes |
| 9 | Mental disorders and psychological problems cannot be prevented | No |
| 10 | Even for severe mental disorders (e.g. schizophrenia), medications should be taken for a given period of time only; there is no need to take them for a long time | No |
| 11 | Positive attitudes, good interpersonal relationships and a healthy life style can help maintain mental health | Yes |
| 12 | Individuals with a family history of mental disorders are at a higher risk for psychological problems and mental disorders | Yes |
| 13 | Psychological problems in adolescents do not influence academic grades | No |
| 14 | Middle-aged or elderly individuals are unlikely to develop psychological problems and mental disorders | No |
| 15 | Individuals with an unstable temperament are more likely to have mental problems | Yes |
| 16 | Mental problems or disorders may occur when an individual is under psychological stress or facing major life events (e.g. death of family members) | Yes |
| 17 | Have you heard about International Mental Health Day? | Yes |
| 18 | Have you heard about the International Day against Drug Abuse and Illicit Drug Trafficking? | Yes |
| 19 | Have you heard about the International Suicide Prevention Day? | Yes |
| 20 | Have you heard about World Sleep Day? | Yes |
